# Physiological and Physical Determinants of Flat-Water Kayaking

**DOI:** 10.3390/muscles4030032

**Published:** 2025-08-14

**Authors:** Yi Shin Lee, Amelia Dingley, Danny Lum, Frankie Tan, John F. T. Fernandes

**Affiliations:** 1 Department of Physical Education and Sport Science, National Institute of Education, Nanyang Technological University, Singapore 639798, Singapore; lysleeyishin@gmail.com; 2 Division of Sport, Health and Exercise Sciences, Brunel University, London UB8 3PH, UK; amelia_dingley@hotmail.com; 3 High Performance Sport Institute, Singapore 397630, Singapore; frankie_tan@sport.gov.sg; 4 Sport Performance and Nutrition Research Group, La Trobe University, Melbourne, VIC 3086, Australia; 5 Department of Physiology, Yong Loo Lin School of Medicine, National University of Singapore, Singapore 119077, Singapore; 6 Cardiff School of Sport and Health Sciences, Cardiff Metropolitan University, Cardiff CF5 2YB, UK; jfernandes@cardiffmet.ac.uk

**Keywords:** anthropometry, muscular strength and power, aerobic capacity, anaerobic power

## Abstract

The main research question of this review involved the identification of the various physiological and physical determinants of flat-water kayaking. A systematic search was conducted using three databases (PubMed, Google Scholar, and Microsoft Academic) between 1991 and December 2022. Quality assessment was performed using a version of the National Heart, Lung and Blood Institute checklist tailored for cross-sectional and observational studies. A total of 169 articles were identified in the initial screening. After duplicate removal and further screening for relevance, a total of 17 articles were included in the review. Altogether, it was found that flat-water kayaking performance was strongly correlated with maximum oxygen uptake (VO_2max_), aerobic and anaerobic thresholds, peak aerobic and anaerobic capacity measure in lab and on the water, and upper and lower-body strength and power, which were measured using heavy resistance, as well as isometric and isokinetic implements. What is less clear is the association between total lean mass and flat-water kayaking performance. However, this may largely be due to the differences in when the measurements were taken.

## 1. Introduction

Sprint kayaking is performed in a straight line over calm water in a one-, two-, or four-person kayak (K1, K2, or K4, respectively), covering distances of 200, 500, and 1000 m [1,2]. Winning performance requires sprint kayakers to produce and maintain the highest average boat velocity over a set distance [3,4]. To achieve this, sprint kayakers must generate high propulsive forces to overcome the drag forces that act upon the kayak to move forward [5]. The winning medal times at the 2020 Olympic games for female K1 over 200 and 500 m distances were 38.120 s and 107.655 s, respectively, while for male K1 over 200 and 1000 m distances were 33.985 s and 200.643 s, respectively [6]. Interestingly, sprint kayakers rarely specialise in one race distance; many race across several distances despite the stark differences in race times [7].

Regardless of race distance, such performance requires the complex integration of physical and physiological components, with each distance requiring a slightly different demand [2]. For example, maximal aerobic power is a greater predictor of 1000 m sprint kayaking performance compared to that of 200 m, while the opposite is true for maximal anaerobic power (i.e., greater prediction for 200 than 1000 m performance) [2]. Despite the clear differences in physiological requirements between sprint kayaking distances, the relationship between upper-body strength and performance is less clear. For example, McKean and Burkett [8] observed weakening relationships between bench press and pull-up one repetition maximum (1RM) and 200, 500, and 1000 m performance over a three-year period in males, while the opposite was true in females. On the other hand, while some studies have demonstrated a relationship between lean body mass and sprint kayaking performance [9], there are other studies that have found this to be untrue [2,10]. Given the seemingly unclear conclusions that have been drawn from the multitude of studies examining the varying aspects of sprint kayaking performance, there is a need to perform a review to unify these findings. An article that reviews the determinants of sprint kayaking performance will help practitioners in adopting the appropriate training regimes. Therefore, our aim is to critically review the physiological and physical determinants of sprint kayaking performance. A secondary aim is to inform future research and provide guidelines for practitioners

## 2. Results

### 2.1. Studies Included in the Review

**Figure 1:**
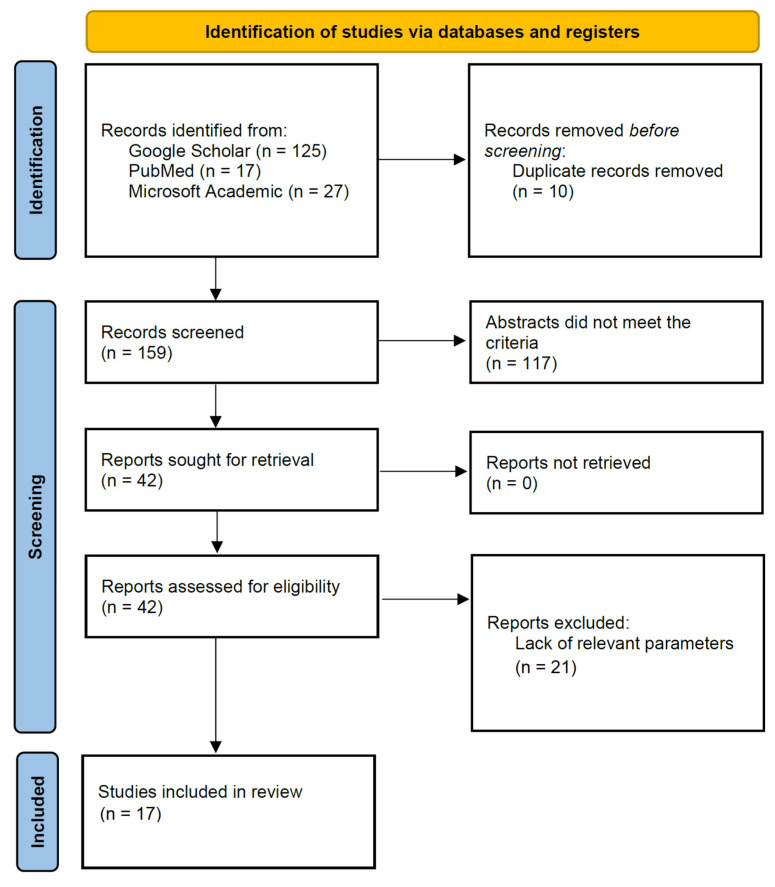
PRISMA flow chart detailing search strategy.

During the initial search, 125 articles were retrieved from Google Scholar, 17 from PubMed, and 27 from Microsoft Academic, resulting in a total of 169 articles. After manually removing duplicates, there were 159 articles left for review. After reviewing the abstracts for relevant studies, 117 studies were excluded, resulting in 42 articles for retrieval. Upon screening the full texts for relevant information, a further 23 articles were excluded, resulting in a total of 17 articles being included for this review. The study was conducted in accordance with the PRISMA checklist. The PRISMA inclusion/exclusion process is graphically represented in Figure 1.

Only observational studies that sought to examine any type of relationship (using correlational or regression statistics) between various types of physiological or physical variables (e.g., aerobic/anaerobic capacity, strength, and muscle girth) and sprint kayaking performance (i.e., on-water or ergometer time trial performance) were included in this review.

In total, we identified 11 studies that investigated the relationship between physiological measures and sprint kayaking performance in 146 participants; 14 studies investigated measures of strength in 376 participants; and 7 explored measures of anthropometry in 280 participants. Post hoc G*Power (G power 3.1.9.7, Dusseldorf, Germany), based on a large effect size (*p* = 0.5), indicated that the power obtained for studies investigating the relationship between physiological measures and sprint kayaking performance ranged from 0.32 to 0.93; studies investigating the relationship between strength measures and sprint kayaking performance ranged from 0.40 to 0.99; and studies investigating the relationship between anthropometry measures and sprint kayaking performance ranged from 0.40 to 0.99.

### 2.2. Quality Assessment Rating

The National Heart, Lung and Blood Institute (NHLBI) checklist produces a qualitative rating (“poor”, “fair”, and “good”) after weighing the various criteria in the paper. Specifically, requiring the critical appraisal of the various criteria laid out in the checklist, the raters came to a conclusion about the quality rating of the study based on how the subjective severity of each criterion influences the study’s overall risk of bias. The checklist does not provide definitive score-to-rating guidelines. Of note, several criteria were not applicable to the cross-sectional study designs used in the studies included in this review (e.g., “Was the time frame sufficient so that one could reasonably expect to see an association between exposure and outcome if it existed?” and “Was loss to follow-up after baseline 20% or less?”). Most of the studies fulfilled the same criteria in general (Table 1); thus, a “poor” rating was only conferred if there was a failure to fulfil a criterion that critically influenced the risk of bias. A total of 4 studies obtained a “good” rating, while 12 studies obtained a “fair” rating. Only one study obtained a “poor” rating. Hence, the current data should be considered to have a low risk of bias.

## 3. Discussion

### 3.1. Physiological Determinants

#### 3.1.1. Metabolic Factors

##### Maximum Oxygen Uptake

Maximal oxygen uptake (VO_2max_) represents the highest capacity that can be used to transport and utilise oxygen during exercise [24]. The mechanisms that underpin VO_2max_ can be grouped into central (i.e., ability of the cardiorespiratory system to transport oxygen) and peripheral factors (i.e., the ability to extract and utilise oxygen) [25]. Refer to the work of Bassett and Howley [24] for a full review. In brief, the central factors pertain to cardiac output and arteriovenous O_2_ difference. On the other hand, peripheral factors pertain to musculoskeletal factors. Though some debate exists, it is generally accepted that central factors limit performance to a greater extent than peripheral factors [24,26]. However, peripheral factors may be influential in exercise and can affect cardiovascular function to improve VO_2max_ [24,27]. Despite the debate in the literature, VO_2max_ is, in general, an indicator of sporting success [28].

Maximal oxygen consumption testing is regularly used to measure aerobic capacity in sprint kayaking. Of the ten available studies that investigated the relationship between VO_2max_ and sprint kayaking performance, nine show a positive inverse relationship (Table 2). Absolute VO_2max_ and VO_2peak_ values have a large to near-perfect [29] inverse correlation with 200 m [−0.59 ≥ *r* ≥ −0.86] [14,21,22], a large to very-large inverse correlation with 500 m [−0.56 ≥ *r* ≥ −0.71] [9,12,16], and a moderate to near-perfect inverse correlation with 1000 m [−0.30 ≥ *r* ≥ −0.90] [14,15,16,18] in relation to sprint kayaking performance. The relationship between relative VO_2max_ and VO_2peak_ values with sprint kayaking performance has been found to be similar. For example, a very large correlation with 200 m [*r* = −0.76] [14], a small-to-very large inverse correlation with 500 m [−0.21 ≥ *r* ≥ −0.82] [12,16,30], and a moderate-to-very large inverse correlation with 1000 m [−0.30 ≥ *r* ≥ −0.84] [14,15,16,17] have been reported within the literature. Only one study has reported opposing findings; Lopez-Plaza et al. [17] found that VO_2max_ was not correlated to 200 m or 1000 m performance [−0.16 ≥ *r* ≥ −0.18]. However, this study estimated VO_2max_ using a multistage shuttle run test (mp3 version, Coachwise, UK), which, given the differences in locomotion between sprint kayaking and running, may not provide a valid assessment of VO_2max_.

Based on the findings of Borges et al. [14] and Fry and Morton [16], it appears that the relationship between VO_2max_ and performance is stronger as the distance of the event increases (Table 2). Given that longer durations have a higher total energy demand and turnover, there is a greater need for aerobic ATP resynthesis. The duration of these longer events (e.g., ~35.197 s, 112.494 s, and 211.447 s for 200 m, 500 m, and 1000 m, respectively) means that the relative contribution from the aerobic pathway is increased and that from the anaerobic pathways is reduced as the distance increases [31]. Those with a higher absolute and relative VO_2max_ can achieve higher rates of ATP resynthesis to fuel performance. Interestingly, even in elite samples, VO_2max_ is still related to performance [22], which practically suggests that all levels who compete at longer distances should seek to improve VO_2max_. For those shorter distances, it is important; however, given the moderate correlations, other factors (e.g., anaerobic) could be involved.

##### Aerobic and Anaerobic Thresholds

The aerobic threshold is often referred to as the lowest intensity at which there is a sustained increase in blood lactate concentration above resting values; this typically occurs between 40 and 60% VO_2max_ [32]. When measured via gas exchange, the aerobic threshold can be defined in three ways—using ventilatory equivalents (intensity where there is a rise in the ventilatory equivalent of O_2_ but not CO_2_), the excess CO_2_ method (intensity where there is an excess CO_2_ production), or the V slope method (intensity where there is a visual inflexion of the minute utilisation of CO_2_ compared to O_2_) [33]. At exercise intensities greater than the aerobic threshold, there is a linear increase in oxygen consumption (VO_2_), carbon dioxide (CO_2_) output, and ventilation. The aerobic threshold has been identified as a predictor of performance in a variety of endurance sports (e.g., running and cycling) and abilities (i.e., trained and untrained) [34,35].

Available data on the relationship between aerobic threshold and sprint kayaking at different distances are limited to two studies [14,16] (Table 3). Both studies concluded that the aerobic threshold has a large inverse correlation with 200 m [*r* = −0.65] [14], 500 m [*r* = −0.48] [16], and 1000 m [−0.63 ≥ *r* ≥ −0.68] [14,16] performance times. The 200 m and 500 m events are of high intensity; hence, they result in the accumulation of high levels of lactate. Therefore, it is plausible that a high aerobic threshold would be favourable as it indicates that athletes would have a higher capacity to buffer hydrogen ions and/or reuse lactate as a fuel [36,37]. Thus, those with a high aerobic threshold can sustain work at the required intensity for a longer period.

While the aerobic threshold has been reported to be a determinant of sprint kayaking performance, it has also been reported that athletes work at an intensity much higher than the aerobic threshold, as indicated by the high level of anaerobic contributions [12]. In view of that, the anaerobic threshold, which is the highest sustained exercise intensity above which production is supplemented by anaerobic mechanisms, may be a more important factor to performance as it is likely closer to the sprint performance exercise intensity.

To the best of our knowledge, only four studies have investigated the relationship between anaerobic threshold and performance times, with all displaying significant correlations (Table 4). The anaerobic threshold has a large to very large correlation with 200 m [−0.54 ≥ *r* ≥ −0.74] [2,14], a large to very large inverse correlation with 500 m [−0.52 ≥ *r* ≥ −0.89] [2,12], and a *very large* inverse correlation with 1000 m performance times [−0.71 ≥ *r* ≥ −0.83] [15].

There is a relationship between anaerobic threshold and performance; anaerobic capacity is still important for longer events as it aids in the high-intensity elements. For instance, overcoming the high inertia at the start, as well as sprint finishes. Similarly to VO_2max_, the relationship between anaerobic threshold and performance times is stronger as the distances increase. This is due to the ability to utilise lactate to resynthesise glucose and the greater capacity to buffer the hydrogen ions, thus allowing athletes to work at a higher intensity for a longer period of time [36,38,39]. Therefore, athletes should look to develop the ability to sustain a high fractional utilisation of their VO_2max_ to become physiologically efficient in sprint kayaking. It is recommended that training at or above the anaerobic threshold intensity is effective in improving the anaerobic threshold in both elite and non-elite individuals [40].

##### Peak Aerobic Power/Anaerobic Capacity

The peak power and anaerobic capacity are two determinants of performance. Peak power represents the maximum instantaneous power that can be achieved during exercise and on a kayak ergometer over a single stroke period [41]. Anaerobic capacity is the maximal amount of ATP resynthesized via anaerobic metabolism during short-duration, maximal exercise [42]. Biological factors such as muscle size, muscle volume, muscle architecture (e.g., pennation angle and fascicle length), neuromuscular activation, and the ability to develop contractile force affect peak power. Consequently, peak power is an important function that underpins the mechanisms of power output [43,44,45,46].

Several studies have investigated the relationship between peak power output on a kayak ergometer and sprint kayaking performance, reporting a *large* inverse correlation with 200 m [−0.68 ≥ *r* ≥ −0.69] [2,10], a *large* to *very large* inverse correlation with 500 m [−0.63 ≥ *r* ≥ −0.84] [2,9], and a *large* inverse correlation with 1000 m kayaking performance [*r* = −0.65] [2] (Table 5). Regarding anaerobic capacity, two studies reported a *very large* inverse correlation between performance times and anaerobic capacity (i.e., 30 s work) [200 m: −0.73 ≥ *r* ≥ −0.74; 500 m: *r* = −0.87; 1000 m: *r* = −0.74) [2,10] (Table 6). With this, fatigue index (i.e., the difference between the peak and minimum power in a Wingate cycle ergometer test) demonstrated a *large* inverse correlation with 200 m and 500 m, respectively [−0.52 ≥ *r* ≥ −0.54] [2]. This relationship is not unexpected given that sprint kayaking also has a large lower-limb contribution [47].

As sprint kayaking requires the locomotion of the athlete’s mass, they require a high relative peak power (i.e., W per kg body mass) and anaerobic capacity [14]. Only one study has identified a relationship between the power-to-weight ratio [*r* = −0.66, *large*] and 1000 m performance [14]. However, this is not as important compared to land-based sports, as the bodyweight is supported by the water. This demonstrates the importance of upper-limb muscular power in kayaking performance [12]. Hence, it is recommended that athletes improve their power in the upper extremities to enhance kayaking performance.

##### Other Metabolic Variables

Skeletal muscle oxygenation may determine sprint kayaking performance-related variables (e.g., cadence) [48]. The tissue saturation index is the difference between the change in deoxyhaemoglobin (i.e., when haemoglobin does not combine with oxygen) and oxyhaemoglobin (i.e., haemoglobin combined with oxygen), which indicates the average oxygen saturation of the muscle. Measuring changes in these muscle oxygenation parameters can be used to gain an insight into muscle metabolism [14].

Two studies have determined the relationship between tissue saturation index and kayaking performance (Table 7). Firstly, Borges et al. [14] observed a *moderate* relationship between tissue saturation index and 200 and 1000 m performance. Paquette et al. [21] found a significantly *very large* to *near-perfect* inverse correlation between 200 m time and the oxygen saturation of the latissimus dorsi, biceps brachii, and vastus lateralis. In addition, a *large* to *very large* correlation between 200 m time and the deoxyhaemoglobin concentration of the latissimus dorsi and vastus lateralis muscles, as well as a combination of deoxyhaemoglobin concentration of the latissimus dorsi, biceps brachii, and vastus lateralis. Furthermore, a *very large* correlation was observed between 500 and 1000 m time and the combined deoxyhaemoglobin concentration of the latissimus dorsi, biceps brachii, and vastus lateralis [21]. In addition, Borges et al. [14] found a *moderate* inverse correlation between 200 m performance and deoxyhaemoglobin concentration, as well as a moderate inverse correlation with 1000 m and deoxyhaemoglobin concentration (see Table 7).

These data indicate that there is greater tissue saturation, higher deoxyhaemoglobin levels, and a greater unloading within the muscles. During the stroke, the forearm muscles are required to perform isometric contractions to hold the paddle, whereas the shoulder complex and muscles such as latissimus dorsi are responsible for the propulsive force in the kayak stroke (specifically at the draw phase of the kayak stroke) [14,49]. These differences may reflect the musculature of the paddle stroke and the ability to rapidly extract oxygen to provide energy to the working muscles. The implication of this may mean that if more haemoglobin is present, more O_2_ can be contained in the body; therefore, the stronger the aerobic capacity. Practically, those working with sprint kayakers need to adopt training and nutritional strategies to maximise haemoglobin mass within their athletes.

### 3.2. Strength and Power

Faster kayak velocities have been attributed to greater stroke rates [47,50], which have been identified as a key indicator of sprint kayaking performance [51]. A higher stroke rate translates to a lower stroke time and thus, a more limited window of time in which force can be produced to propel the kayak. As such, it is critical that the athlete produces the greatest amount of force possible in the short interval of each stroke in order to optimise sprint kayaking performance. This requires a high impulse and rate of force development (RFD). Maximal strength capacity, which is the ability to produce the maximal amount of external force [52], has been positively correlated with RFD [53,54,55], highlighting the importance of strength in sprint kayaking performance. Power, which is the product of force and velocity, has also been linked to higher RFD [56], warranting investigating into how power correlates with sprint kayaking performance in the literature.

#### 3.2.1. Upper-Body Pulling Capacity

The majority of studies that have investigated the relationship between sprint kayaking performance and muscular strength have typically measured upper-body pulling and pushing strength. It was found that there was a *moderate* to *very large* relationship between upper-limb pulling and pushing strength and sprint kayaking performance, whether the metric was time trial (TT) performance across multiple distances [pulling: −0.49 ≥ *r* ≥ −0.85; pushing: −0.53 ≥ *r* ≥ −0.97] or power output on a kayak ergometer [pulling: 0.83 ≤ *r* ≤ 0.86; pushing: 0.59 ≤ *r* ≤ 0.74]. These are consolidated in Table 8.

Studies examining the relationship between upper-body pulling strength and on-water sprint kayaking TT duration across various race distances have found *large* to *very large* inverse correlations, whether it was with the bench pull [−0.68 ≥ *r* ≥ −0.85] [11,15], pull-up [−0.59 ≥ *r* ≥ −0.85], isokinetic pulling power and strength in a simulated kayak stroke [−0.47 ≥ *r* ≥ −0.66] [2,10], or isometric measures of upper-body pulling strength [−0.70 ≥ *r* ≥ −0.80] [23]. Further, isometric measures have also been associated with a greater kayak ergometer power output [0.83 ≤ *r* ≤ 0.86] [19]. It was further reported in another study that the change in isometric bench pull peak force after a period of strength training was positively correlated to the change in 200 m kayak ergometer power output [*r* = 0.52] [19]. This suggests that isometric strength measures may not just be a suitable alternative to dynamic strength measures when examining transfer to sprint kayaking performance, but that isometric strength itself is a variable that warrants development in sprint kayakers.

Given this wealth of evidence, it is clear that there is a strong role for upper-body pulling strength in sprint kayaking performance. This is unsurprising due to the pulling contribution in horizontal arm action during a paddle stroke [57] that is required to produce an adequate amount of propulsive force to overcome the drag forces acting on the moving kayak and accelerate the boat. However, beyond maximum strength, strength endurance is also critical for sprint kayaking performance, given the multiple strokes that need to be produced consistently over the course of the race. Akca and Muniroglu [11] found that performance in a 1 min bench pull test at 40% 1RM was inversely correlated with 200 m [*r* = −0.71], 500 m [*r* = −0.85], and 1000 m [*r* = −0.65] TT durations. These findings were supported by Broďáni et al. [13], who also found that a test of power endurance in the bench pull was inversely correlated with 200 m [*r* = −0.51] and 500 m [*r* = −0.49] TT durations, demonstrating that the strength endurance of the upper-body pulling muscles is also key in sprint kayaking performance. This makes sense given that sprint kayak events are at least 30 s in duration [6] and require multiple kayak strokes to be produced cyclically over the duration of the race. Given the strength and volume of these data, it is important that practitioners make a concerted effort to improve the upper-body pulling capacities of their athletes, whether through dynamic and/or isometric means.

#### 3.2.2. Upper-Body Pushing Capacity

Existing evidence also clearly indicates a strong role of upper-body pushing strength in sprint kayaking performance. Several studies have demonstrated a *large* to *near-perfect* inverse correlation between 1RM and 3RM bench press strength and TT durations across 200 m [−0.56 ≥ *r* ≥ −0.85], 500 m [−0.55 ≥ *r* ≥ −0.91], and 1000 m [−0.62 ≥ *r* ≥ −0.97] distances [8,16,22], as well as a *large* correlation with 120 s all-out kayak ergometer performance [*r* = 0.59] [9]. Interestingly, Akca and Muniroglu [11] did not find any correlation between 1RM bench press strength and TT duration. This is possibly owing to the non-standard bench press technique (i.e., keeping the lower back in contact with the bench) that was employed, and the participants may not have been confident with maximal exertion in such a position. Nevertheless, all other studies that measured 1RM or 3RM bench press strength found an inverse correlation with TT duration across multiple distances. In addition to dynamic strength measurements, isometric bench press peak force and RFD were inversely correlated with 200 m sprint kayak TT duration [−0.53 ≥ *r* ≥ −0.75], as well as being positively correlated with kayak ergometer mean power output [0.64 ≤ *r* ≤ 0.79] [19]. Altogether, these findings provide strong evidence in relation to the importance of upper-body pushing strength in sprint kayaking performance.

These data are of no surprise given the upper-body kinematics of paddling outlined in a study by Mann and Kearney [57], where they described both a push and pull component of horizontal arm action during a paddle stroke. The ability to produce a greater pushing strength should translate to a greater force production in the aforementioned push component and thus, a better sprint kayaking performance. Further, Akca and Muniroglu [11] found that performance in a 1 min bench press test at 40% 1RM was inversely correlated with 200 m [*r* = −0.80], 500 m [*r* = −0.89], and 1000 m [*r* = −0.72] TT durations, suggesting the importance of upper-body strength endurance in sprint kayaking performance. Surprisingly, Broďáni et al. [13] found that there were no correlations between tests of maximum power and power endurance in the bench press and the 200 m, 500 m, and 1000 m events. However, it must be noted that the study procedures were unclear. That is, the loading conditions and process for selecting loads were not reported. This is important as the load has a direct impact on the magnitude of the power value during pushing and pulling exercise [58]. Moreover, the sprint kayaking times were taken from an unknown period between 2019 and 2021 (the corresponding author was contacted but no reply was received). Thus, the results must be interpreted with caution.

#### 3.2.3. Lower-Body Capacity

While there is a greater relative abundance of research demonstrating correlations between upper-body pulling and pushing strength and sprint kayaking performance, it does not discount the importance of lower-body strength on sprint kayaking performance. Isometric squat peak force has been found to have a *moderate* to *large* correlation with 200 m TT duration [−0.44 ≥ *r* ≥ −0.67] and 200 m kayak ergometer power output [0.47 ≤ *r* ≤ 0.61] [19]. Moreover, Pickett et al. [22] found that isometric mid-thigh pull force at 0.03 s was correlated with 200 m TT duration [*r* = −0.49], while Hamano et al. [9] found that isokinetic knee flexion strength was positively correlated with the average power produced during a 120 s all-out kayak ergometer test [0.71 ≤ *r* ≤ 0.72]. It was also reported that the change in isometric squat peak force after a period of training was positively correlated to the change in 200 m ergometer kayaking power output [*r* = 0.52] [20]. While Pickett et al. [22] reported no significant relationship between deadlift strength and sprint kayaking performance, this may have been due to the 2 min rest periods between maximal attempts, which may have been too short [59,60] and likely reduced maximal force production in subsequent sets, thus potentially hindering the expression of a more accurate 1RM. Additionally, López-Plaza et al. [17,18] found that countermovement jump performance was inversely correlated with 200 m [*r* = −0.23], 500 m [*r* = −0.39], and 1000 m [*r* = −0.23] TT durations, indicating the importance of lower-body power in sprint kayaking performance. These are consolidated in Table 9.

Together, these studies provide evidence for the importance of lower-body strength and power in enhancing sprint kayaking performance. This is unsurprising, given how the greater involvement of leg musculature during kayaking is one of the differentiating factors between international-, national-, and club-level kayakers [47], as well as there being evidence to show that lower-limb force production is responsible for up to 21% of the force produced during a paddle stroke and 16% of the average kayak speed [61]. While there are far fewer studies that have shown a positive association between lower-body strength and power and sprint kayaking performance, this is largely due to the dearth of studies that have investigated this relationship in the first place, with only 5 out of the 12 studies in this review having conducted lower-body strength and/or power tests.

While it was shown in Table 2 that the relationship between VO_2max_ and performance is stronger as the distance of the event increases, the magnitude of the relationship between strength measures and kayaking performance distance is distinct. For example, Akca and Muniroglu [11] reported that the relationship between strength measures and 500 m kayaking performance was higher than that observed for 200 m and 1000 m events. This was supported by the findings of van Someren and Howatson [2], who also reported a higher correlation between strength measures and 500 m kayaking performance compared to 200 m kayaking performance. However, McKean and Burkett [8] reported that strength measures had the highest correlation with 1000 m kayaking performance after three years of training. One possible reason for the current observation could be that 200 m kayakers, in general, place a lot of focus on developing maximum strength, as the event requires them to generate a high amount of force in order for the boat to travel fast over the short distance. Hence, kayakers in this event would likely have similar strength statuses. In this case, performance for the 200 m event would then have to a greater dependence on other factors. On the other hand, kayakers in longer-distance events may have placed more emphasis on developing their aerobic and anaerobic capacity and less on strength development. Hence, kayakers with greater strength levels may then be able to have an edge over their competitors.

In conclusion, there is a large amount of evidence to suggest that both upper- and lower-body strength are key in sprint kayaking performance. As such, significant emphasis should be placed on strength training. Strength training has been shown to contribute positively to increases in RFD [62,63], with one study showing that maximal strength may account for up to 80% of the variance in RFD in the 150–250 ms range [64]. If increases in maximal strength are sought, strength training should be performed at higher relative intensities, i.e., 80% and above [65,66].

### 3.3. Physical Determinants

#### 3.3.1. Anthropometry

Athletes’ anthropometric characteristics might also influence performance in sprint kayaking. In sprint kayaking, distinct anthropometry has been linked to higher-level performances. For example, elite sprint kayakers tend to be taller and heavier than their sub-elite counterparts [16,67,68]. Similarly, successful male kayakers have a certain morphology and anthropometric attributes [10,16]. As such, their stature, arm span, leg length, and arm fat mass have a positive relationship with their performance [9]. Whilst this information is important for applied practitioners working with sprint kayakers, there is little information on how these anthropometric characteristics differ between race distances. Indeed, most studies have simply provided anthropometric characteristics between distinctive levels of performance. In addition, these data were identified almost 20 years ago and there is a need to pull this information together. It is important as some talent identification programmes, e.g., handball, futsal, and rhythmic gymnastics, base some characteristics such as stature as a determinant to identify talented athletes [69,70,71]. Furthermore, a recent study identified and included stature and body mass within their identification model, and anthropometry made up 16% of the priority in relation to identifying talent in sprint kayaking athletes [72]. Importantly, readers should be aware that only body mass is modifiable; if sprint kayakers are aiming to increase body mass, then this should be conducted by accruing lean body mass. Therefore, practitioners may wish to monitor body composition variables alongside changes in body mass.

#### 3.3.2. Muscle Girth and Mass

Muscle girth and fibre size have been positively correlated with force production [73]. An increased muscle girth theoretically translates to an increase in sarcomere formation and thus the potential for the formation of more actin–myosin cross-bridges, allowing for a higher force production. However, it must be noted that force production has both neural and muscular components, and a larger muscle girth does not necessarily translate to a greater ability to produce force, especially when compared across different training populations [74,75]. That said, the comparison may be more appropriate for relatively homogenous well-trained populations [76,77] such as across groups of kayakers. This is consolidated in Table 10.

Multiple studies have found an inverse correlation between the girths of the upper limbs and chest with sprint kayaking TT duration [−0.42 ≥ *r* ≥ −0.80] [2,10,11,16], as well as a positive correlation with kayak ergometer power output [*r* = 0.83] [9]. Of note, the nature of most anthropometric measurements would mean that bicep girth also includes the triceps, while a chest girth measurement would also include the muscles of the back, such as the latissimus dorsi. The triceps and chest are heavily involved in upper-body pushing action, while the biceps and latissimus dorsi are heavily involved in upper-body pulling action. As mentioned above, these are actions that are heavily involved in the propulsion of the kayak [57]. As such, it appears that a better muscular development of these areas is associated with a better sprint kayaking performance.

Further, lower-limb girths have been both inversely correlated with sprint kayaking TT duration [−0.77 ≥ *r* ≥ −0.81] [11] and positively correlated with kayak ergometer power output [*r* = 0.91] [9]. Once again, this is expected given the earlier established importance of lower limb force production on sprint kayaking performance [47,61]; increased muscular development in these areas should lead to a better sprint kayaking performance.

Lastly, trunk girth has been positively correlated with kayak ergometer power output [0.74 ≤ *r* ≤ 0.75] [9]. This association is expected given the high and constant activation of the trunk musculature throughout a paddling cycle [47], as well as a notational analysis demonstrating the importance of trunk actions in sprint kayaking performance. Considered in conjunction with the earlier established relationship between muscle girth and force production in a relatively homogenous training population, it is expected that better-developed muscles would aid performance through enhanced strength and power.

On the other hand, the correlation between total lean body mass and sprint kayaking performance is less clear. While Hamano et al. [9] found that total, arm, leg, and trunk lean mass was positively correlated with kayak ergometer power output [0.64 ≤ *r* ≤ 0.90], two other studies investigating the relationship between lean body mass and on-water sprint kayaking TT duration did not [2,10]. A possible explanation could be the method by which lean body mass was derived (dual-energy x-ray absorptiometry scans vs. skinfold analysis). Another possible explanation could be the difference in the way sprint kayaking performance was determined (kayak ergometer in the work of Hamano et al. [9] vs. on-water kayaking in the works of van Someran and Howatson [2] and van Someran and Palmer [10]). Testing on the kayak ergometer does not account for technical competency such as the ability to maintain equilibrium on the boat. Another possibility could be the differences in the period of their macrocycle in which the participants were tested. As short-term gains in myofibrillar hypertrophy likely have a negligible impact on force production [78], a lack of correlation between lean mass and sprint kayaking performance could potentially be due to individuals being tested earlier in their off-season. Given the lack of clarity and the dearth of the current body of evidence, more work has to be carried out in this area to ascertain the relationship between lean body mass and sprint kayaking performance before we can draw any definitive conclusions.

Nonetheless, there is sufficient evidence to suggest that upper- and lower-limbs as well as trunk, girths are positively associated with sprint kayaking performance, indicating that it may be prudent to dedicate a certain portion of the athletes’ training sessions towards muscular hypertrophy. While muscle hypertrophy generally occurs across a broad range of intensities [79], it is important to note that sets should generally be taken closer to failure than if the goal were solely maximal strength [65]. Further, most individuals have a limited amount of time to train and may be better served by performing sets on the heavier side in order to shorten the amount of time taken to reach failure, concurrently fulfilling the dual role of developing higher force production capabilities.

### 3.4. Gaps in the Research

Based on the results of this review, there are a limited number of studies that have investigated the metabolic requirements for successful sprint kayaking performance. A comprehensive assessment of the metabolic requirements and determinants of sprint kayaking performance will support athletes when programming training regimens. Therefore, future studies should look to do this, especially pertaining to work surrounding the lactate threshold, e.g., transition from a heavy to a severe domain, which is particularly lacking.

The relationship between VO_2max_ and anaerobic threshold with race performance is stronger as the race distance increases. However, the relationship between peak power, anaerobic capacity, aerobic threshold, and anaerobic power reserve to race distance is unclear and requires establishment [80]. This is especially important, given that sprint kayakers rarely specialise in one race distance. Establishing the relationship between physiological and physical qualities with race distance will support sprint kayakers when looking to track performance over time [81], as well as in planning and programming their training. Therefore, there is a gap in research relating to identifying how an athlete should train in order to be successful over a range of race distances over time. Whilst correlational and cross-sectional data are useful, researchers should employ more robust interventional methodologies.

When discussing the literature concerning the relationship between strength and sprint kayaking performance, there is a plethora of studies suggesting the importance of both upper-body pulling and pushing capacity in improving sprint kayaking performance and kayak ergometer power. However, this abundance of research does not apply to the relationship between lower-body capacity and sprint kayaking performance. Given that the lower body theoretically plays an important role in sprint kayaking performance based on its kinematics, there is a need for further research exploring the effects of lower-body muscle function (e.g., lower-body strength and the rate of force production) on said performance. Another point to take note of is that only one study included in the review investigated the relationship between strength measures and kayaking performance specifically in females, with results showing a small correlation [18]. The strength measures used were mainly medicine ball throws, which differed from the traditional strength measurements (e.g., 1RM, isometric, and isokinetic). Furthermore, the participants were young female athletes that might not have developed technical proficiency yet. Hence, more studies investigating the relationship between maximal strength (e.g., 1RM, isometric, and isokinetic peak force) and sprint kayaking are required to better understand the influence of muscular strength on sprint kayaking performance in female athletes.

Even the relationship between lean mass and sprint kayaking performance is unclear. There are almost just as many studies that have shown a positive correlation as there are those that do not. It might be worthwhile to consider longitudinal studies carried out over an entire competition period (between the off-season and competitive season), where correlations between lean mass and sprint kayaking performance are constantly monitored. Furthermore, using a long-term athletic development approach, researchers and/or practitioners could track changes in lean mass and the effect this has on performance. A final consideration to make is that there may be some point of diminishing returns and/or a negative effect of increasing lean mass on sprint kayaking performance, as it is likely to affect relative strength and power—a relationship that may be more pertinent with longer-distance races.

### 3.5. Practical Recommendations

From the review, several variables that are key for sprint kayaking performance have been identified. Specifically, it seems that aerobic and anaerobic thresholds, VO_2max_, 1RM pull-up and bench press strength, and muscle girths were all highly correlated with sprint kayaking performance across 200, 500, and 1000 m race distances; thus, improving these would be key for enhancing said performance.

From a practical standpoint, practitioners may monitor the maximal aerobic power (MAP) of the athletes. This variable can be used as an indication of the athletes’ cardiovascular function. The MAP profiles will have to be determined individually and specifically for on-water performances [80,82]. Similarly, given the importance of upper-body strength in sprint kayaking performance, maximal strength testing should be performed early on in the training cycle. Practitioners may adopt either 1RM or isometric tests for bench presses, bench pulls, and squats to monitor the strength development of the athletes.

Practitioners may use the MAP obtained to determine the appropriate training intensity to improve the athletes’ anaerobic and aerobic capacities. Each individual should train at an appropriate percentage of MAP and work–rest ratios to elicit favourable changes in either anaerobic or aerobic capacities—the focus of which will depend on what the individual lacks, as well as their training phase within the periodization cycle. Practitioners may also determine the strength training loads based on individual 1RM values. The appropriate weights should match the varying intensities across the athlete’s training macrocycle, with the overarching goal of being able to produce as much force as possible in both upper- and lower-body exercises.

## 4. Materials and Methods

### Search Strategy

The studies included for review in this paper were gathered according to the Preferred Reporting Items for Systematic reviews and Meta-analyses (PRISMA) guidelines [83]. The literature search and verification were conducted by the two lead authors (A.D. and Y-S.L.)

A systematic search of three databases was performed (PubMed, Google Scholar, and Microsoft Academic). Only articles published in English were considered. Using Boolean logic, databases were searched to identify articles containing the following keywords and combinations:

(a) ‘kayaking’ OR ‘sprint’ OR ‘flat water’ OR ‘kayaking’ OR ‘water-sport’ OR ‘kayak’ OR ‘kayak ergometer’;

(b) ‘VO2’ OR ‘maximal oxygen uptake’ OR ‘oxygen consumption’ OR ‘lactate threshold’ OR ‘economy’ OR ‘anaerobic power’ OR ‘aerobic capacity’ OR ‘power at VO_2max_’ OR ‘VO2 at lactate threshold’ OR ‘metabolic accumulation’ OR ‘blood lactate’ OR ‘anaerobic threshold’ OR ‘critical power’ OR Critical speed’ OR ‘acceleration’ OR ‘arm span’ OR ‘sitting height’ OR ‘height’ OR ‘stature’ OR ‘body mass’ OR ‘flexibility’ OR ‘strength’ OR ‘endurance’ OR ‘anthropometry’ OR ‘physiological’ OR ‘physical’ OR ‘power’ OR ‘maximal power’ OR ‘power:weight ratio’ OR ‘skinfold’ OR ‘fat-free mass’ OR ‘lean mass’ OR ‘body composition’ OR ‘fat mass’.

## 5. Conclusions

There is a seemingly unclear conclusion in previous studies examining the determinants of sprint kayak performance; our aim was to critically review the physiological and physical determinants of sprint kayak performance in order to support future research and provide guidelines for practitioners. Our review of the existing literature has highlighted the need for clarity with notable gaps, with insights for future research and practical application. As such, the strong relationship between VO_2max_ and anaerobic threshold and race performance, with increasing race distances, highlights the importance of these determinants in shaping the competitive outcomes for sprint kayak performance. However, given that the relationship between peak power, anaerobic capacity, aerobic threshold, and anaerobic power reserve and race distance is unclear highlights the need for further research. The call for establishing these relationships, as identified by Sandford et al. [80] is especially pertinent given the multifaceted nature of sprint kayaking, where athletes often compete across various race distances. Understanding how physiological and physical qualities interplay with race distance is imperative not only for tracking performance over time, as highlighted by Winchcombe et al. [81], but also for informing the design of effective training programmes that can enhance the overall performance across an array of race distances.

This review also underscores the need for a more nuanced exploration of the relationship between lower-body muscle function and sprint kayaking performance. While upper-body pulling and pushing capacity have been extensively studied, the lower body’s role remains less clear, despite its theoretical importance in the kinematics of sprint kayaking. Future research should investigate the effects of lower-body muscle function, such as the strength and rate of force production, on overall performance.

The complex and inconclusive nature of the relationship between lean mass and sprint kayaking performance requires further investigation, with considerations for longitudinal studies spanning entire competition periods. Monitoring the correlations between lean mass and performance over time, as well as adopting a long-term athletic development approach, could provide a clearer understanding of how changes in lean mass may impact performance. Additionally, the exploration of potential diminishing returns or negative effects of increasing lean mass on performance, particularly in longer-distance races, adds a layer of complexity that warrants further attention.

Moving forward, researchers and practitioners should strive for more robust interventional methodologies to complement correlational and cross-sectional data. By addressing these research gaps, we can better guide the training and development of sprint kayakers, ultimately optimising their performance across a variety of race distances, as well as advancing our understanding of the intricate interplay between physiological and physical determinants in relation to sprint kayaking.

## Figures and Tables

**Table 1:** Characteristics and NHLBI checklist rating of studies included in the review.

Authors	n	Sex	Population (Kayakers)	NIH Rating
Akca et al. [11]	11	M	National level	Good
Bishop [12]	9	F	High performance	Fair
Broďáni et al. [13]	17	M and F	International level	Poor
Borges et al. [14]	21	M and F	Well trained; junior	Fair
Forbes et al. [15]	13	M and F	Junior	Fair
Fry et al. [16]	38	M	State and non-state level	Fair
Hamano et al. [9]	2	M	College; well trained	Fair
López-Plaza et al. [17]	89	M	Young	Good
López-Plaza et al. [18]	86	F	Young	Fair
Lum et al. [19]	23	M and F	Well trained	Good
Lum et al. [20]	20	M and F	National and collegiate level	Good
McKean et al. [8]	24	M and F	Elite level	Fair
Paquette et al. [21]	19	M	Highly trained	Fair
Pickett et al. [22]	22	M	National and international level	Fair
Uali et al. [23]	10	M and F	Elite level; junior	Fair
van Someran et al. [10]	26	M	National and international level	Fair
van Someran et al. [2]	18	M	Competitive	Fair

**Table 2:** Relationship between VO_2max_ and VO_2peak_ with sprint kayaking performance.

Authors	n	Sex	Population	200 m	500 m	1000 m
Bishop [12]	9	F	High performance	-	−0.72 to −0.82	-
Borges et al. [14]	21	M and F	Well-trained junior	−0.76 to −0.86	-	−0.84 to −0.90
Forbes et al. [15]	18	M and F	Junior	-	-	−0.76 to −0.90
Fry and Morton [16]	38	M	State and non-state	-	−0.56 to −0.59	−0.64 to −0.71
Hamano et al. [9]	12	M	College; well trained	-	0.63	-
López-Plaza et al. [18]	86	F	Young	-	-	−0.31
López-Plaza et al. [30]	89	M	Young	-	−0.22	-
Paquette et al. [21]	19	M and F	Highly trained	0.75	-	-
Pickett et al. [22]	22	M	National/international	−0.71	-	-

**Table 3:** Relationship between aerobic threshold and sprint kayaking performance.

Authors	n	Sex	Population	200 m	500 m	1000 m
Borges et al. [14]	21	M and F	Well-trained juniors	−0.65	-	−0.68
Fry and Morton [16]	38	M	State and non-state	-	−0.48	−0.63

**Table 4:** Relationship between anaerobic threshold and sprint kayaking performance.

Authors	n	Sex	Population	200 m	500 m	1000 m
Bishop [12]	9	F	High performance	-	−0.89	-
Borges et al. [14]	21	M and F	Well-trained juniors	−0.74	-	-
Forbes et al. [15]	18	M and F	Juniors	-	-	−0.71 to −0.83
van Someran and Howatson [2]	18	M	Competitive	−0.54	−0.52	-

**Table 5:** Relationship between peak power and sprint kayaking performance.

Authors	n	Sex	Population	200 m	500 m	1000 m
Hamano et al. [9]	12	M	College; well trained	-	0.63	-
van Someran and Howatson [2]	18	M	Competitive	−0.68	−0.84	−0.65
van Someran and Palmer [10]	26	M	National and international	−0.69	-	-

**Table 6:** Relationship between anaerobic capacity and sprint kayaking performance.

Authors	n	Sex	Population	200 m	500 m	1000 m
van Someran and Howatson [2]	18	M	Competitive	−0.74	−0.87	−0.74
van Someran and Palmer [10]	26	M	National and international	−0.73	-	-

**Table 7:** Relationship between tissue saturation index and sprint kayaking performance.

Authors	n	Sex	Population	200 m	500 m	1000 m
Borges et al. [14]	21	M and F	Well-trained junior kayakers	−0.42 to −0.54	−0.49	−0.49
Paquette et al. [21]	22	M	National/international	−0.70 to −0.93	−0.85	−0.85

**Table 8:** Relationship between upper-body capacity and sprint kayaking performance.

Authors	n	Sex	Population	200 m	500 m	1000 m	Kayak Ergometer
Akca and Muniroglu [11]	11	M	National	−0.80 (1 min bench press) −0.68 (1RM bench pull) −0.71 (1 min bench pull)	−0.89 (1 min bench press) −0.80 (1RM bench pull) −0.85 (1 min bench pull)	−0.72 (1 min bench press) −0.65 (1 min bench pull)	-
Broďáni et al. [13]	17	M and F		−0.51 (power endurance test for bench pull)	−0.49 (power endurance test for bench pull)	-	-
Forbes et al. [15]	13	M and F	Junior	-	-	−0.92 (1RM bench press) −0.85 (1RM bench pull)	-
Fry and Morton [16]	38	M	State and non-state	-	−0.46 to −0.62 (isokinetic simulated kayak stroke)	−0.55 to −0.68	-
Hamano et al. [9]	12	M	College; well trained	-	0.63	-	0.64 (grip strength) 0.59 (1RM bench press)
López-Plaza et al. [17]	89	M	Young	0.51 (overhead medicine ball throw)	0.44 (overhead medicine ball throw)	0.51 (overhead medicine ball throw)	
López-Plaza et al. [18]	86	F	Young	0.29 (overhead medicine ball throw)	0.22 (overhead medicine ball throw)	0.28 (overhead medicine ball throw)	
Lum and Aziz [19]	23	M and F	Well-trained	−0.70 to −0.75 (isometric bench press peak force) −0.53 to −0.61 (isometric bench press RFD) −0.83 to −0.88 (isometric-prone bench pull peak force) −0.53 to −0.62 (isometric-prone bench pull RFD)	-	-	0.74 to 0.79 (isometric bench press peak force) 0.64 to 0.68 (isometric bench press RFD) 0.83 to 0.86 (isometric-prone bench pull peak force) 0.66 to 0.86 (isometric-prone bench pull RFD)
Lum et al. [20]	20	M and F	National and collegiate	-	-	-	0.52 (isometric bench pull peak force @120 degrees)
McKean and Burkett [8]	25	M and F	Elite	−0.56 to −0.85 (1RM bench press) −0.63 to −0.82 (1RM pull-up)	−0.55 to −0.91 (1RM bench press) −0.60 to −0.82 (1RM pull-up)	−0.62 to −0.97 (1RM bench press) −0.59 to −0.85 (1RM pull-up)	-
Pickett et al. [22]	22	M	National/international	−0.80 (3RM bench press) −0.76 (3RM bench row) −0.73 (3RM chin-up)	-	-	-
van Someren and Howatson [2]	18	M	Competitive	−0.47 (isometric pulling strength in simulated kayak stroke) −0.57 (isokinetic pulling power in simulated kayak stroke)	−0.60 (isometric pulling strength in simulated kayak stroke) −0.66 (isokinetic pulling power in simulated kayak stroke)	-	-
van Someren and Palmer [10]	26	M	National/international	−0.47 (isokinetic pulling power in simulated kayak stroke)			

**Table 9:** Relationship between lower-body capacity and sprint kayaking performance.

Authors	n	Sex	Population	200 m	500 m	1000 m	Kayak Ergometer
Hamano et al. [9]	12	M	College; well trained	-	-	-	0.71 to 0.72 (isokinetic knee flexion)
López-Plaza et al. [18]	86	F	Young	−0.23 (counter movement jump)	-	-	-
López-Plaza et al. [17]	89	M	Young	−0.23 (counter movement jump)	−0.39 (counter movement jump)	−0.23 (counter movement jump)	-
Lum and Aziz [19]	23	M and F	Well trained	−0.44 to −0.67 (isometric squat peak force) −0.47 (isometric squat RFD)	-	-	0.47 to 0.61 (isometric squat peak force)
Lum et al. [20]	20	M and F	National and collegiate	−0.51 (isometric squat peak force @90 degrees)	-	-	-
Pickett et al. [22]	11	M and F	Well trained	0.05 (IMTP peak) −0.49 (IMTP @0.03 s)	-	-	-

**Table 10:** Relationship between lean mass and/or muscle girths and sprint kayaking performance.

Authors	n	Sex	Population	200 m	500 m	1000 m	Kayak Ergometer
Akca and Muniroglu [11]	11	M	National	−0.70 (relaxed biceps girth) −0.80 (flexed biceps girth)	−0.76 (relaxed biceps girth) −0.80 (flexed biceps girth) −0.81 (thigh girth)	−0.77 (thigh girth)	-
Fry and Morton [16]	38	M	State and non-state	-	−0.49 (biceps girth) −0.42 (forearm girth) −0.52 (chest girth)	−0.64 (biceps girth) −0.60 (forearm girth) −0.68 (chest girth)	-
Hamano et al. [9]	12	M	College; well trained	-	-	-	0.73 (upper-limb girth) 0.62 (lower-limb girth) 0.83 (chest girth) 0.74 (waist girth) 0.75 (hip girth) 0.91 (calf girth) 0.90 (arms lean mass) 0.85 (legs lean mass) 0.64 (trunk lean mass) 0.82 (total body lean mass)
López-Plaza et al. [18]	86	F	Young	−0.35 (muscle mass)	−0.34 (muscle mass)	−0.32 (muscle mass)	-
López-Plaza et al. [17]	89	M	Young	-	−0.24 (muscle mass percentage)	-	-
van Someren and Howatson [2]	18	M	Competitive	−0.53 (chest girth)	−0.50 (chest girth)		
van Someren and Palmer [10]	26	M	National/international	−0.41 (tensed upper-arm girth) −0.44 (relaxed upper-arm girth) −0.48 (tensed forearm girth) −0.51 (relaxed forearm girth) −0.51 (chest girth)	-	-	-

## Data Availability

No new data were created or analyzed in this study. Data sharing is not applicable to this article.

## Abbreviations

The following abbreviations are used in this manuscript:

1RM	1 repetition maximum
3RM	3 repetition maximum
ATP	Adenosine triphosphate
VO_2max_	Maximal oxygen uptake
